# Obesity is associated with heavy menstruation that may be due to delayed endometrial repair

**DOI:** 10.1530/JOE-20-0446

**Published:** 2021-03-09

**Authors:** Jane J Reavey, Catherine Walker, Alison A Murray, Savita Brito-Mutunayagam, Sheona Sweeney, Moira Nicol, Ana Cambursano, Hilary O D Critchley, Jacqueline A Maybin

**Affiliations:** 1 MRC Centre for Reproductive Health, University of Edinburgh, Edinburgh, UK

**Keywords:** endometrium, menses, inflammation, obese, menorrhagia, AUB

## Abstract

Heavy menstrual bleeding is common and debilitating but the causes remain ill defined. Rates of obesity in women are increasing and its impact on menstrual blood loss (MBL) is unknown. Therefore, we quantified BMI and MBL in women not taking hormones and with regular menstrual cycles and revealed a positive correlation. In a mouse model of simulated menstruation, diet-induced obesity also resulted in delayed endometrial repair, a surrogate marker for MBL. BrdU staining of mouse uterine tissue revealed decreased proliferation during menstruation in the luminal epithelium of mice on a high-fat diet. Menstruation is known to initiate local endometrial inflammation and endometrial hypoxia; hence, the impact of body weight on these processes was investigated. A panel of hypoxia-regulated genes (*VEGF*, *ADM*, *LDHA*, *SLC2A1*) showed consistently higher mean values in the endometrium of women with obesity and in uteri of mice with increased weight vs normal controls, although statistical significance was not reached*.* The inflammatory mediators, *Tnf* and *Il6* were significantly increased in the uterus of mice on a high-fat diet, consistent with a pro-inflammatory local endometrial environment in these mice. In conclusion, obesity was associated with increased MBL in women. Mice given a high-fat diet had delayed endometrial repair at menstruation and provided a model in which to study the influence of obesity on menstrual physiology. Our results indicate that obesity results in a more pro-inflammatory local endometrial environment at menstruation, which may delay endometrial repair and increase menstrual blood loss.

## Introduction

Abnormal uterine bleeding (AUB) is a common and incapacitating symptom that affects up to one in three women of reproductive age (RCOG 2011). Heavy menstrual bleeding (HMB) is one of the most common reasons for referral to gynaecology clinics with greater than 800,000 women seeking treatment per year in the United Kingdom alone (NICE 2018). In addition, HMB has a significant economic impact. A conservative estimation of the cost of menstrual complaints from the United States revealed that each woman with HMB spends $333 per year on extra menstrual and pharmaceutical products. Furthermore, indirect costs due to work absence or inability to perform childcare/household tasks resulted in a loss of $2291 per woman per year (Frick *et al.* 2009). These figures are in addition to the financial costs generated in general practice and specialist hospital services. Subjectively, HMB is defined as excessive menstrual blood loss which interferes with a woman’s physical, social, emotional and/or material quality of life (NICE 2018). An objective definition is a total menstrual blood loss (MBL) of greater than 80 mL per menstrual cycle.

According to the FIGO classification system, abnormal uterine bleeding may be categorised into structural causes (PALM: Polyps, Adenomyosis, Leiomyoma, Malignancy and hyperplasia) and non-structural causes (COEIN: Coagulopathy, Ovulatory dysfunction, Endometrial, Iatrogenic and Not otherwise classified) (Munro *et al.* 2011, 2018). Up to 50% of women have no structural cause for their regular AUB and are assigned the AUB-E (AUB of endometrial origin) category. The cause of AUB-E remains undefined but current studies implicate excessive inflammation, delayed repair of the endometrium and/or impaired vasoconstriction of the specialised endometrial spiral arterioles (Marsh *et al.* 1997, Abberton *et al.* 1999, Malik *et al.* 2006, Critchley *et al.* 2020).

Obesity is an abnormal or excessive fat accumulation that presents a risk to health and is quantified as a BMI of ≥30 kg/m^2^ (see WHO Fact Sheet 'Obesity and overweight' website: https://www.who.int/news-room/fact-sheets/detail/obesity-and-overweight (accessed 27 August 2020)). Data from the Health Survey for England showed that the prevalence of obesity in women aged 35–44 was 24% in 2009, 30% in 2018 and has increased to 33% in 2019 (NHS 2020). Obesity has previously been identified as having a profound impact on female reproductive health. High BMI is associated with early initiation of menarche, menstrual irregularities during adolescence, polycystic ovary syndrome, suboptimal hormonal contraceptive efficacy and infertility (Zaadstra *et al.* 1993, Pettigrew & Hamilton-Fairley 1997, Lash & Armstrong 2009, Kulie *et al.* 2011). Adipose tissue is a major endocrine organ and adipose-derived hormones may have a significant impact on uterine/endometrial function and thus influence on quantity of MBL. Minimal data are available in the literature to determine the influence of BMI on volume of menstrual blood loss, making it difficult to counsel women appropriately in the clinical setting.

We hypothesised that a high BMI would result in increased menstrual blood loss and contribute to the symptom of HMB. To test this hypothesis, 121 women completed a pictorial based assessment chart (PBAC) of their menstrual loss and calculated their BMI. To decrease genetic and environmental heterogeneity, we also used a mouse model of simulated menstruation (Brasted *et al.* 2003) where mice were randomised to a high-fat or control diet for three months prior to menses induction. As a surrogate marker of MBL, endometrial repair was graded and compared in the two groups (Kaitu’u-Lino *et al.* 2007). Current evidence indicates that hypoxia is required for efficient endometrial repair in both women and the mouse model of simulated menses (Maybin *et al.* 2018) and that increased endometrial inflammation is associated with HMB (Malik *et al.* 2006). Therefore, a panel of hypoxia regulated genes and inflammatory mediators were compared in human and mouse tissue, comparing results in normal and high BMI/weight groups.

## Methods

### Human studies

**Figure 1:**
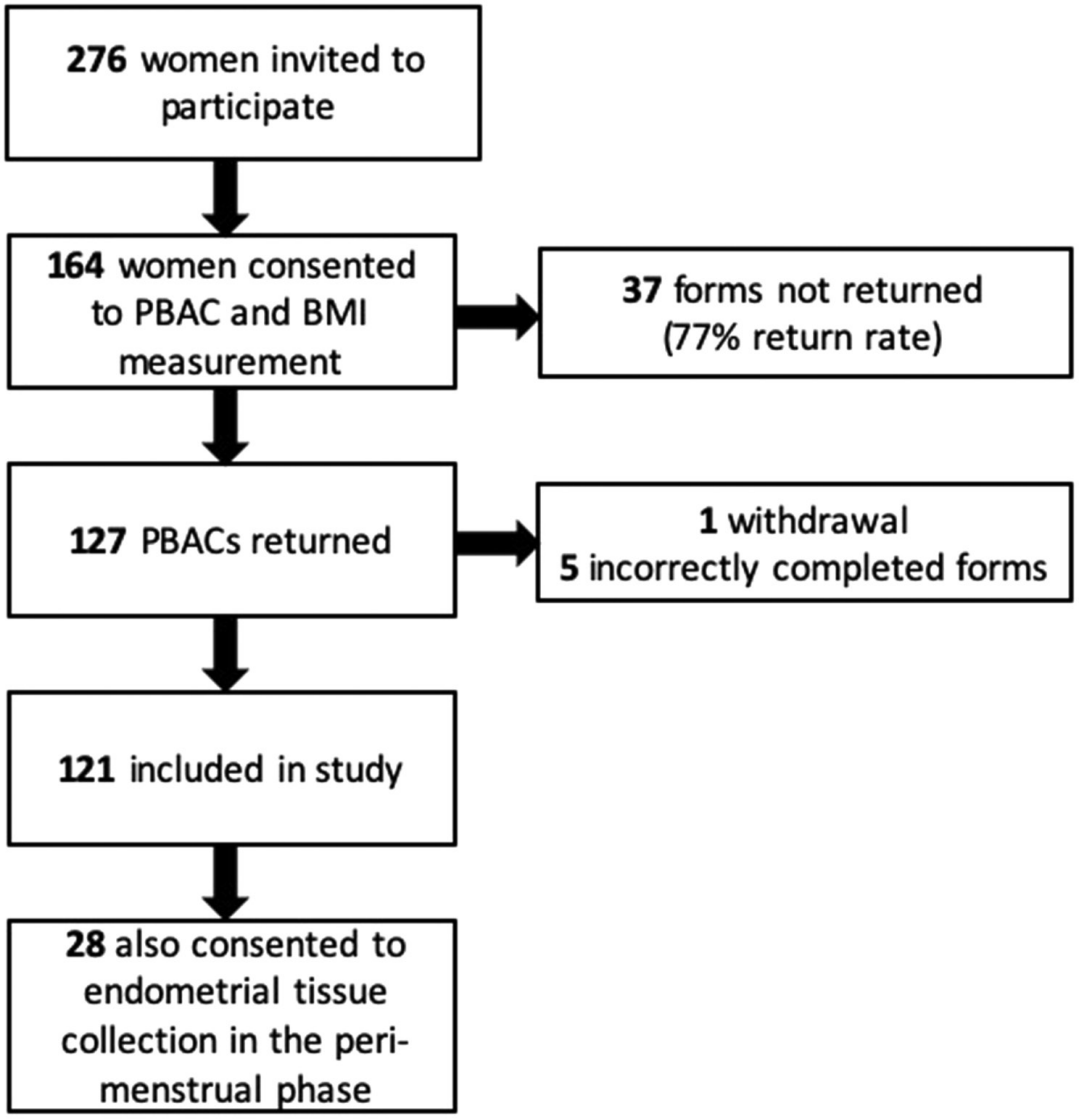
Participant recruitment. PBAC, pictorial-based assessment chart.

Height and weight measurement were obtained from 164 healthy women of reproductive age attending gynaecological outpatient clinics in NHS Lothian. Written consent was obtained from all participants and a favourable ethical opinion granted from Lothian Research Ethics Committee (REC 15/WS/0212, REC 10/S1402/59, REC 07/S1103/29, REC 1994/6/17). All women reported regular menstrual cycles (21–35 days). The women were not using hormonal contraceptives and had no exogenous hormone exposure for 2 months prior to participation. In this study, 121 women returned a fully completed pictorial-based assessment chart (PBAC; Fig. 1) (Table 1). The PBAC contains pictorial representation of graded staining from slight to severe, across different absorbencies of menstrual towels and tampons. This is a validated technique and correlates with objective menstrual blood loss measurements obtained using the alkaline haematin method (Wyatt *et al.* 2001). Participants were asked to complete the PBAC each time they changed their menstrual pad/tampon over one menses. A scoring system for the PBAC was based on previous studies to give an estimated MBL in millilitres (Higham *et al.* 1990, Wyatt *et al.* 2001).

**Table 1:** Summary of characteristics of 121 participants completing the pictorial-based assessment chart (PBAC) as mean (range) or number (%).

Characteristics of the participants	
Age	42.8 (19–55)
Parity	1.6 (0–4)
Body mass index	26.9 (17.2–43.6)
Fibroids on ultrasound	
Present	48 (39.7%)
Absent	58 (47.9%)
Unknown	15 (12.4%)
Fibroid size (cm)*	4.4 (0.5–11.8)
Smoking (never)	50 (41.3%)
Use of mefenamic acid/tranexamic acid during PBAC	42 (34.7%)
Diabetes	1 (0.82%)

*Measurements only in women with fibroids present.

Endometrial biopsies (*n*  = 28) were collected during the late secretory or menstrual phase with a suction curette (Pipelle, Laboratorie CCD, Paris, France) from a subset of participants without fibroids >3 cm or symptoms of endometriosis (Table 1). Twenty women had a BMI <30 and 8 a BMI >30. Tissue was divided and (i) placed in RNA later, RNA stabilization solution (Ambion (Europe) Ltd., Warrington, UK), (ii) fixed in 4% neutral buffered formalin for wax embedding. Biopsies were confirmed as late secretory/menstrual by (i) histological dating (criteria of Noyes *et al.* 1950), (ii) reported last menstrual period and (iii) serum progesterone and oestradiol concentrations at the time of biopsy (Table 2).

**Figure 2:**
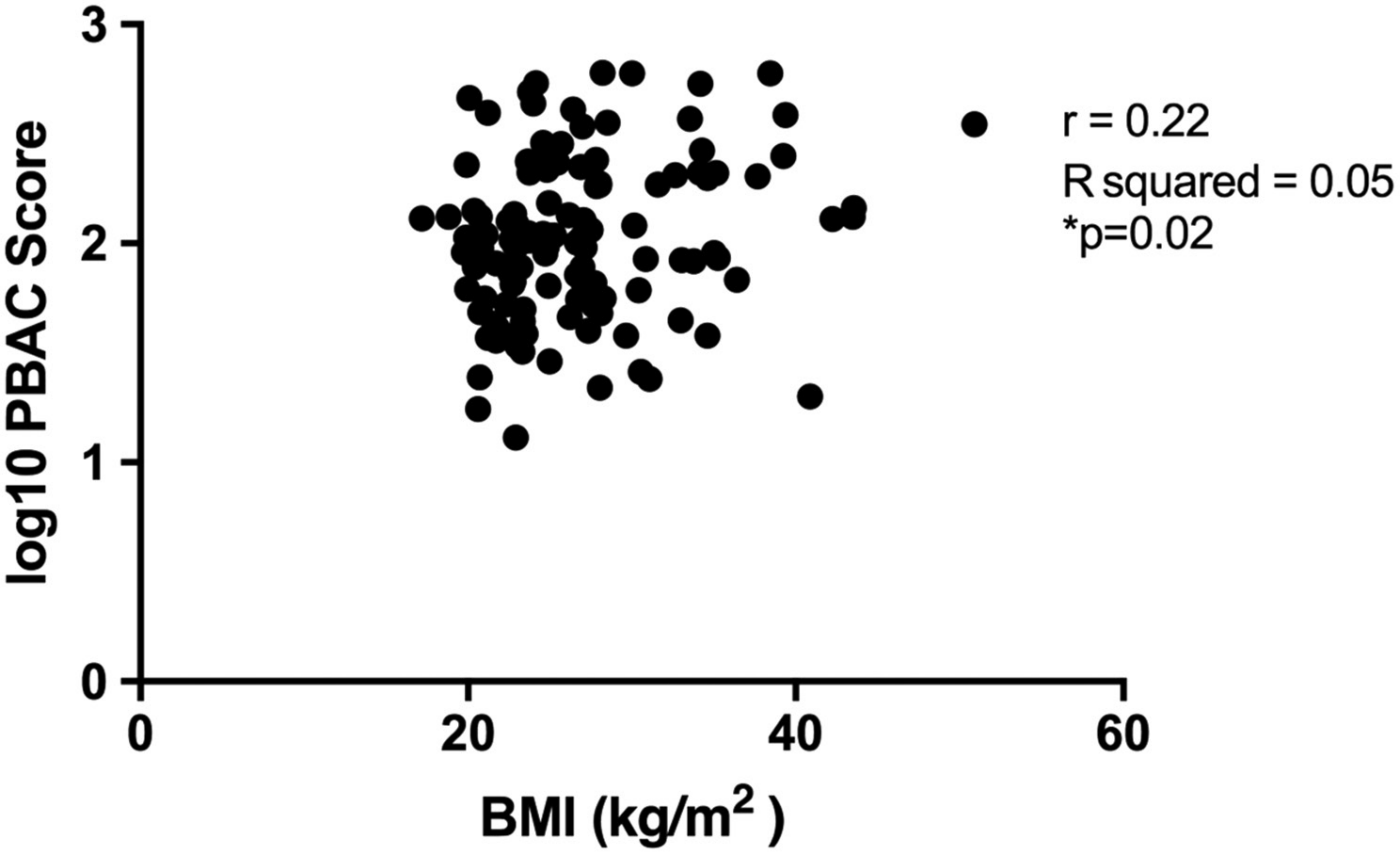
Association between human participant BMI and menstrual blood loss (MBL). Linear correlation analysis of BMI and MBL assessed by pictorial-based assessment chart (PBAC) score.

**Table 2:** Serum hormone levels in women providing endometrial biopsies.

	Non-obese (BMI<30, *n* = 20)	Obese (BMI>30, *n* = 8)
Mean BMI, kg/m^2^ (range)	22.6 (20.6–28.9)	37.1 (33.3–42.3)
Mean serum oestradiol, pmol/L (range)	247.5 (20–1142)	258.3 (78–1177)
Mean serum progesterone, nmol/L (range)	8.37 (0.2–18.9)	2.2 (0.2–3.4)

Menstrual blood loss (MBL) was objectively measured in the women providing endometrial tissue using a modified alkaline haematin method (Hallberg & Nilsson 1964, Maybin *et al.* 2017) and heavy menstrual bleeding (HMB) defined as a blood loss of >80 mL per cycle. Women were given the same brand of menstrual products (Tampax® tampons/Always® towels, Proctor & Gamble, UK) with verbal and written instruction on collection. The technique was validated in our laboratory using a known volume of whole blood applied to menstrual products. The proportion of women with HMB in those with a BMI <30 and >30 who provided endometrial biopsies was 60% and 50%, respectively.

### Mouse studies

All experimental animal procedures were approved by the University of Edinburgh ethical committee and performed in accordance with the Animals Scientific Procedures Act (1986) of the UK Home Office (PPL 70/8754). Female C57BL/6JOlaHsd mice were purchased from Envigo (Hillcrest, UK). Mice were randomised to high fat or control diet (Special Diets Services 826172 – RM 58% AFE Fat or 826171 – RM 11% AFE Fat) for 12 weeks and body weight recorded weekly. Endometrial shedding and repair were simulated in ovariectomised mice as previously described (Brasted *et al.* 2003, Cousins *et al.* 2014). In brief, 6- to 9-week-old female mice were ovariectomised on day 1 of the protocol to deplete endogenous steroid production. Mice then received daily subcutaneous injections of oestradiol (E_2_) in peanut oil (100 ng) on days 7–9. A progesterone implant (P4) was placed subcutaneously on day 13, mice also received daily injections of E_2_ (5 ng) from days 13 to 15. On day 15, decidualisation of one uterine horn was induced by transcervical injection of 20 μL peanut oil using a non-surgical transfer device (ParaTechsTM, Lexington, KY, USA). Decidualisation is a prerequisite for endometrial breakdown at menses. P4-withdrawal was induced 4 days after decidualisation (day 19) by removal of the P4 implant to trigger endometrial breakdown and repair. Mice received an intra-peritoneal injection of bromodeoxyuridine (BrdU, 0.25 mg) 1.5 h prior to culling. Mice were culled by cervical dislocation 24 h after P4-withdrawal (T24). Uteri were dissected and collected in RNA later (Ambion) and 4% neutral buffered formalin for paraffin embedding. Any animal with failed decidualisation was excluded from the study as there was no endometrial breakdown/repair to grade. This was *n*  = 2 out of 12 mice fed a normal diet (16.6% failed decidualisation rate) and *n*  = 9 out of 15 mice on a high fat diet (60% failed decidualisation rate).

### Endometrial repair histological grading

5μm mouse uterine sections were stained with haematoxylin and eosin (H&E) and stage of breakdown/repair graded by two masked independent observers using a previously published scoring system (*n*  = 11 normal diet, *n*  = 6 high-fat diet) (Kaitu’u-Lino *et al.* 2007, 2009, Maybin *et al.* 2018) (Table 3). Very occasionally a section showed features between two grades and this was assigned a score of x.5. The average score from the two reviewers was used to grade endometrial repair in each mouse. Where scores differed by >2 grades, the observers examined the slide together and decided upon the most appropriate score. Statistical analysis was carried out on the finalised histological scores. The data are displayed as a percentage of mice at each repair grade and values were rounded to the nearest whole, with values of >0.5 rounded up.

**Table 3:** Histological scoring system for endometrial repair (Kaitu’u-Lino *et al.* 2007).

Histological repair grade	Histological description
1	Decidualised tissue. Expansion of stromal compartment, presence of decidual cells. Glands pushed towards the myometrium.
2	Early breakdown. Some loss of structural integrity between decidual cells. Most decidua still intact.
3	Complete breakdown. Complete tissue destruction in decidual zone. No intact decidual tissue. Some sloughing of the endometrium from the myometrium.
4	Early repair. Beginnings of re-epithelialisation. Dissociation of necrotic tissue from the myometrium.
5	Complete repair. Stromal restoration, complete re-epithelialisation. Small amount of luminal debris.

### Immunohistochemistry

Endometrial cell proliferation was detected in 5 μm mouse uterine sections using BrdU antibody. Paraffin sections were dewaxed and rehydrated. Heat induced antigen retrieval was carried out in 0.1 M citrate buffer (pH 6) in a pressure cooker. Endogenous peroxidase activity was quenched by immersing slides in 3% hydrogen peroxide in methanol. Sections were sequentially incubated in avidin and biotin (Vector, Burlingame, CA, USA) according to manufacturer’s instructions. Normal rabbit serum was used as protein block (30 min) and as diluent for primary and secondary antibodies. BrdU antibody (Fitzgerald, Acton, MA, USA) was applied (1:5000) and sections incubated overnight at 4°C. Negative controls were incubated with Sheep IgG (Merck, Dorset, UK) at the same concentration as the primary antibody. Biotinylated rabbit anti-sheep secondary antibody (Vector) was applied at 1:200 for 30 min. Sections were incubated with avidin-biotin-peroxidase complex (Vector) before visualisation of positive signal using diaminobenzidine (DAKO, Santa Clara, CA, USA). Sections were counter-stained with hematoxylin, dehydrated and mounted with Pertex (Cellpath, Hemel Hempstead, UK). The images were taken using a Zeiss Z1 imager widefield microscope (×20 magnification), captured by Axiocam HRC camera and processed using ZEN software.

### Real-time quantitative reverse transcription PCR

Total RNA from human endometrium and mouse uterine samples was extracted using the RNeasy Mini Kit (Qiagen Ltd) according to manufacturer’s instructions. RNA samples were reverse transcribed using iScript cDNA synthesis kit (Bio-Rad Laboratories Ltd) alongside control samples. mRNA transcripts were quantified relative to appropriate reference genes (Human: *SDHA* and *ATP5B*, Mouse: *RLP13* and *ACTB*), as determined by geNorm assay (Primerdesign Ltd, Southampton, UK). Specific primers were designed using the universal probe library assay design centre and checked with BLAST (Table 4). Reactions were performed in triplicate alongside controls using ABI QuantStudio system under standard conditions with TaqPath ProAmp Master Mix (Life Technologies). Quantification was performed using the 2-ΔΔCt method with normalisation against a sample of liver or placental cDNA.

**Table 4:** Details of PCR primers and universal probe library probe number.

Gene of interest	Primer (forward)	Primer (reverse)	Probe
*VEGFA*	CATTGGAGCCTTCCCTTG	ATGATTCTGCCCTCCTCCTT	22
*ADM*	GCCTGCCCAGACCCTTAT	GTAGCGCTTGACTCGGATG	57
*LDHA*	TCTCTGTAGCAGATTTGGCAGA	AAGACATCATCCTTTATTCCGTAAA	31
*SLC2A1*	GGTTGTGCCATACTCATGACC	CAGATAGGACATCCAGGGTAGC	67
*IL10*	TGGGGGAGAACCTGAAGAC	CCTTGCTCTTGTTTTCACAGG	30
*IL6*	GATGAGTACAAAAGTCCTGATCCA	CTGCAGCCACTGGTTCTGT	40
*Il1B*	TACCTGTCCTGCGTGTTGAA	TCTTTGGGTAATTTTTGGGATCT	78
*TNF*	TCCAGACTTCCTTGAGACACG	CCCGGTCTCCCAAATAAATAC	36
*Vegfa*	TTAAACGAACGTACTTGCAGATG	AGAGGTCTGGTTCCCGAAA	4
*Adm*	TTCGCAGTTCCGAAAGAAGT	AGCGAGTCCCGTAGGGTAG	42
*Ldha*	GGCACTGACGCAGACAAG	TGATCACCTCGTAGGCACTG	12
*Slc2a1*	GGACCCTGCACCTCATTG	GCCACGATGCTCAGATAGG	20
*Il10*	CAGAGCCACATGCTCCTAGA	TGTCCAGCTGGTCCTTTGTT	41
*Il6*	GCTACCAAACTGGATATAATCAGGA	CCAGGTAGCTATGGTACTCCAGAA	6
*Il1b*	AGTTGACGGACCCCAAAAG	AGCTGGATGCTCTCATCAGG	38
*Tnf*	CTGTAGCCCACGTCGTAGC	TTTGAGATCCATGCCGTTG	25

### Statistical analysis

Statistical analysis was performed using the GraphPad Prism 8 Software version 8.4.3 (GraphPad Prism Software, Inc.). Since the MBL values were highly skewed, the association between MBL and BMI was assessed by linear regression using the logarithms of the MBL values as the response variable. Forward stepwise multiple linear regression was used to test which other factors significantly predicted the logarithm of MBL after adjusting for the effect of BMI. Mann–Whitney tests were used to analyse mouse endometrial repair and human and mouse PCR data. A value of *P <* 0.05 was considered statistically significant.

## Results

### There is a positive correlation between BMI and menstrual blood loss measured by PBAC score

Heavy menstrual bleeding, defined as a PBAC score >80 mL, was present in 63% of participants. Women with obesity (BMI >30) constituted 25% of all participants. Regression analysis showed a weak positive association between menstrual pictorial-based assessment chart (PBAC) score and BMI (Fig. 2, *P* = 0.02). Multiple regression showed that only the presence of fibroids added significantly to BMI in predicting PBAC (*P* = 0.004), with BMI remaining borderline significant when adjusted for fibroids (*P* = 0.051). Among other potential confounders, after adjusting for BMI and fibroids, the *P*-values were, respectively, 0.43 for age, 0.57 for parity, 0.08 for smoking and 0.14 for mefenamic or tranexamic acid. Given the known heterogeneity of these human participants, including age, parity, presence of fibroids and genetic variations (Table 1), we used the mouse model of simulated menstruation.

### Mice with high body weight had delayed endometrial repair

**Figure 3:**
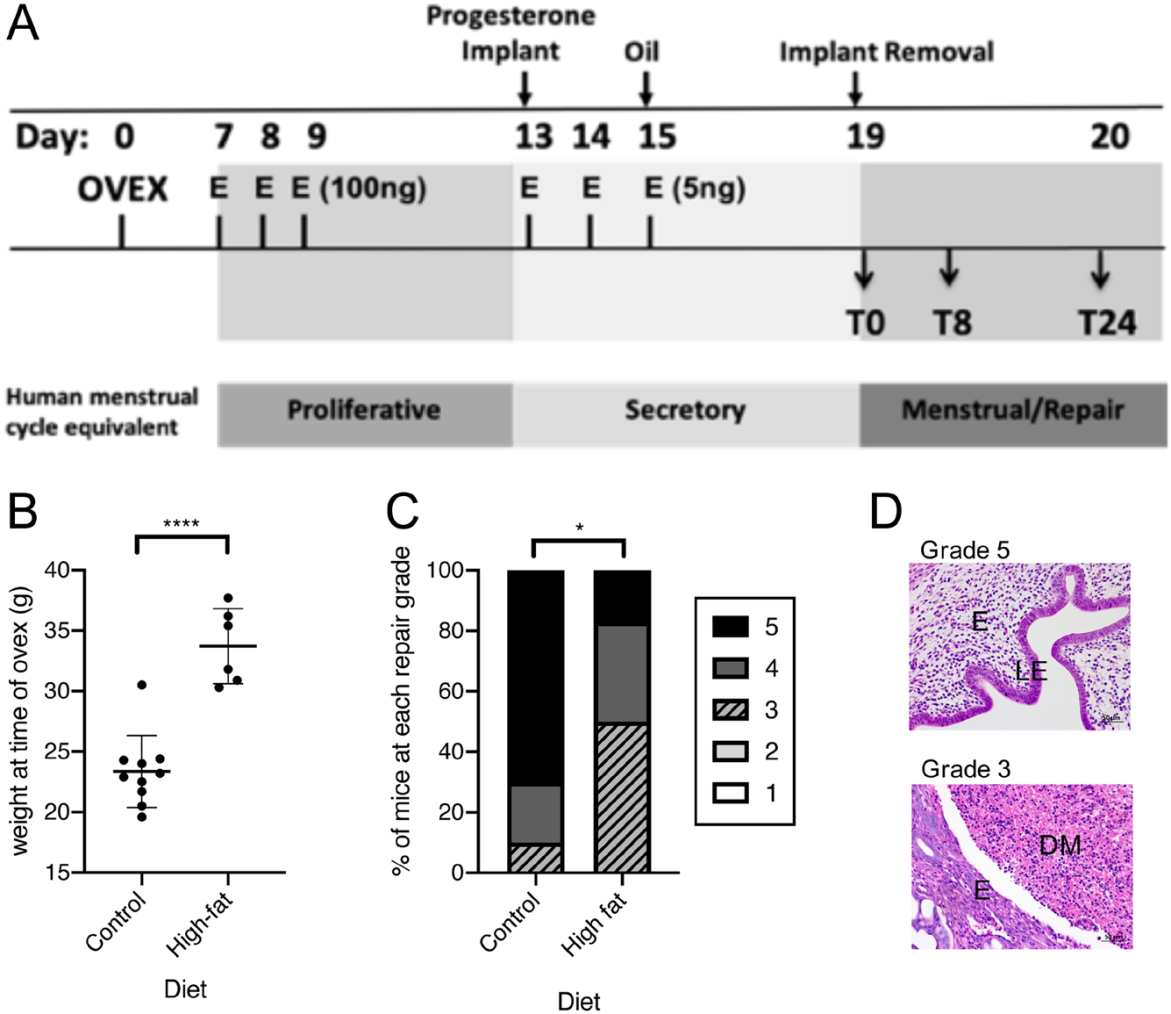
A high fat diet resulted in delayed endometrial repair in the mouse model of menstruation. (A) Mouse model of simulated menstruation. E, oestradiol, Ovex ovariectomy. T0 time of progesterone implant removal, T8, 8h following progesterone withdrawal, T24, 24h following progesterone withdrawal. (B) Mice maintained on a high-fat diet for 3 months prior to menses induction had significantly increased body weight at the time of ovariectomy when compared to those on normal diet. (C) Mice on a high-fat diet had significantly decreased histological repair grades 24 h following progesterone withdrawal. The graph shows the percentage of mice at each repair grade. (D) Representative images of histological repair grade 5 (LE, luminal epithelium fully restored) and grade 3 (DM, decidualised mass present, minimal epithelial coverage). E, endometrium. ****P< 0.0001, *P < 0.05.

Women with HMB are known to bleed for longer than those with normal loss (Maybin *et al.* 2018), consistent with delayed endometrial repair. As determination of menstrual blood loss is difficult and often inaccurate in mice, we quantified endometrial repair as a surrogate marker for HMB. Mice were placed on a high fat or normal diet prior to induction of simulated menstruation to define the role of weight on endometrial repair during menstruation (Fig. 3A).

Mice on a high fat diet (*n*  = 6) had a significantly increased body weight (*P* < 0.0001) with a mean weight of 34 g vs a mean weight of 23 g in mice maintained on a normal diet (*n*  = 10) (Fig. 3B). Quantification of endometrial breakdown and repair from grade 1 (decidualisation) to 5 (full repair) was assessed at 24 h following progesterone withdrawal. This timepoint was selected as endometrial repair has previously been shown to be well underway by 24 h after removal of the progesterone pellet (the trigger for menstruation). This revealed that mice on a high fat diet had significantly delayed endometrial repair vs those on a normal diet (mean 3.5 vs 4.4, *P* < 0.05). The percentage of mice at each endometrial repair grade is shown in Fig. 3C and representative images of histological grade 5 (full repair) and grade 3 (complete breakdown), the most common grade assigned in the normal and high-fat diet groups respectively, are shown in Fig. 3D.

### Mice with high body weight had decreased endometrial cell proliferation 24h following progesterone withdrawal

**Figure 4:**
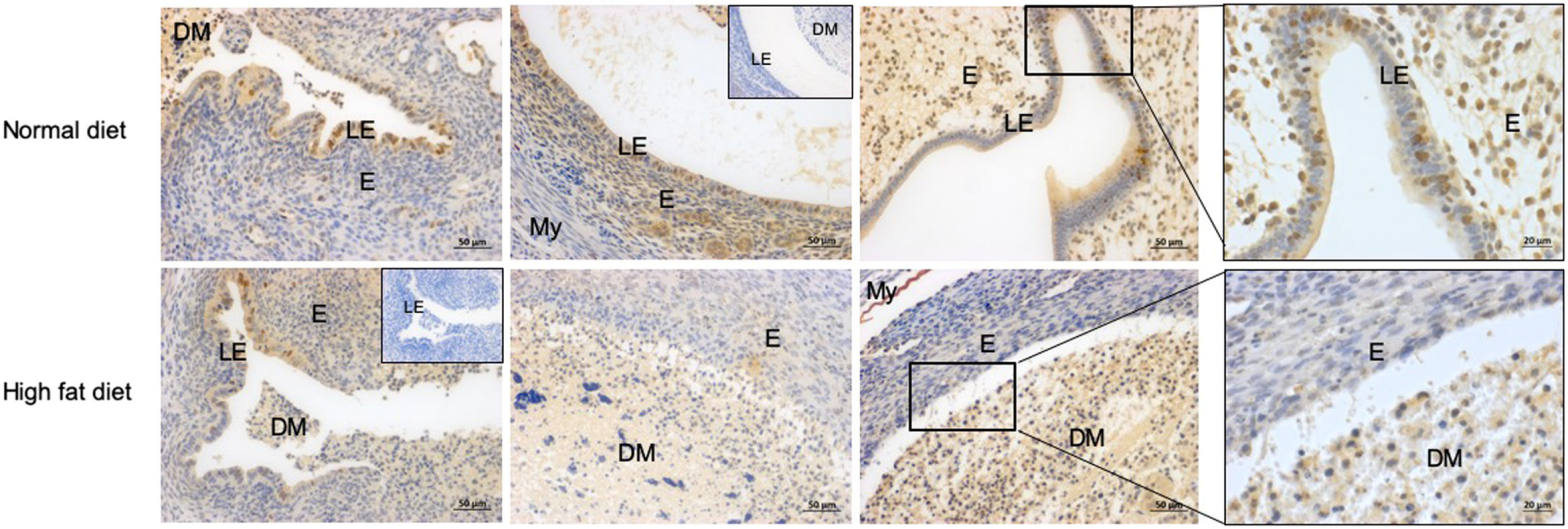
Mice on a high fat diet have decreased endometrial BrdU staining 24 h following progesterone withdrawal. Upper panel = representative slides from three mice on normal diet. Lower panel = representative slides from three mice on a high fat diet. Images on right of each panel = higher magnification. LE, luminal epithelium; DM, decidualised mass; E, endometrium; My, myometrium; insets, negative control stained with concentration matched IgG.

Progesterone withdrawal is the trigger for menstruation and we have previously determined that 24h following progesterone implant removal in our mouse model is the time of active endometrial repair (Maybin *et al.* 2018). To examine the impact of body weight on endometrial cell proliferation at menstruation, immunohistochemical staining for BrdU was carried out on uterine sections collected 24 h following progesterone withdrawal. This revealed positive nuclear staining in luminal epithelial cells and occasional stromal cells (Fig. 4). Comparison of staining from mice on a normal vs high-fat diet showed increased BrdU staining at 24 h in mice maintained on a normal diet (Fig. 4).

### Impact of obesity on hypoxia regulated genes

**Figure 5:**
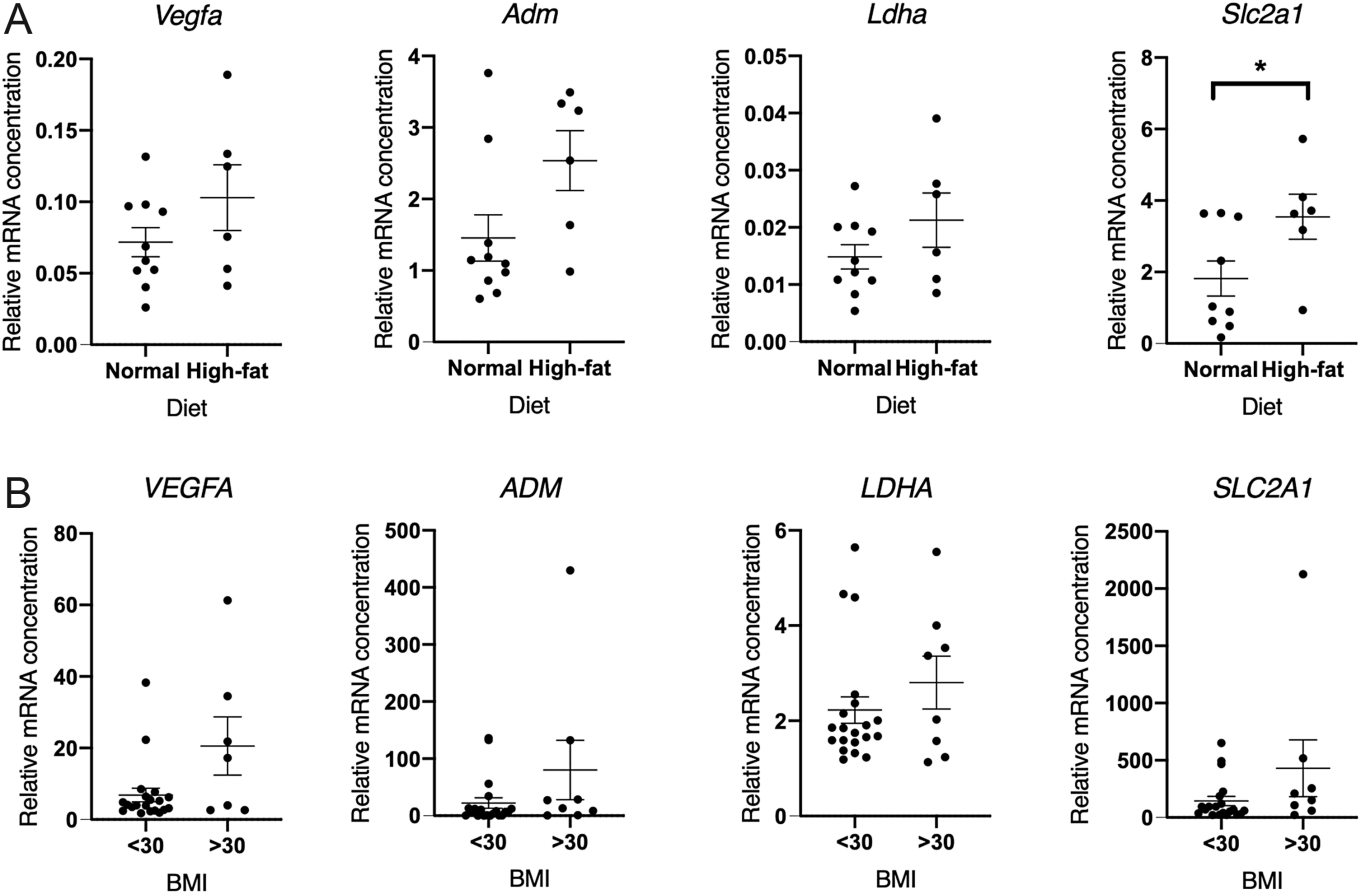
Reverse transcription quantitative real-time PCR assessment of hypoxia-regulated genes. (A) *Vegfa*, *Adm*, *Ldha* and *Slc2a1* concentrations in decidualised uterine tissue from mice on normal (*n*  = 10) and high-fat (*n*  = 6) diets 24 h following progesterone withdrawal. (B) *VEGFA*, *ADM*, *LDHA* and *SLC2A1* concentrations in late secretory and menstrual endometrial tissue from women with a normal BMI (<30, *n*  = 20) and those who are classified as having obesity (>30, *n*  = 8). Mean and s.e.m. mean displayed on graphs, *P < 0.05.

We have previously shown that a lack of hypoxia delays endometrial repair (Maybin *et al.* 2018). Therefore, we investigated the impact of high body weight on a panel of known hypoxia-regulated genes in uterine samples from mice and endometrium from women in the late secretory/menstrual phase without fibroids >3 cm (Fig. 5A). Mice with high body weight had significantly increased uterine* Slc2a1* 24 h following progesterone withdrawal compared to those of normal weight (*P*< 0.05) but this increase was not observed for Vegfa, Adm or Ldha. In women, there were no significant differences in late secretory/menstrual phase endometrial *VEGFA*, *ADM*, *LDHA* or *SLC2A1* between obese women and those with a normal BMI (Fig. 5B).

### Impact of obesity on local uterine inflammatory markers

**Figure 6:**
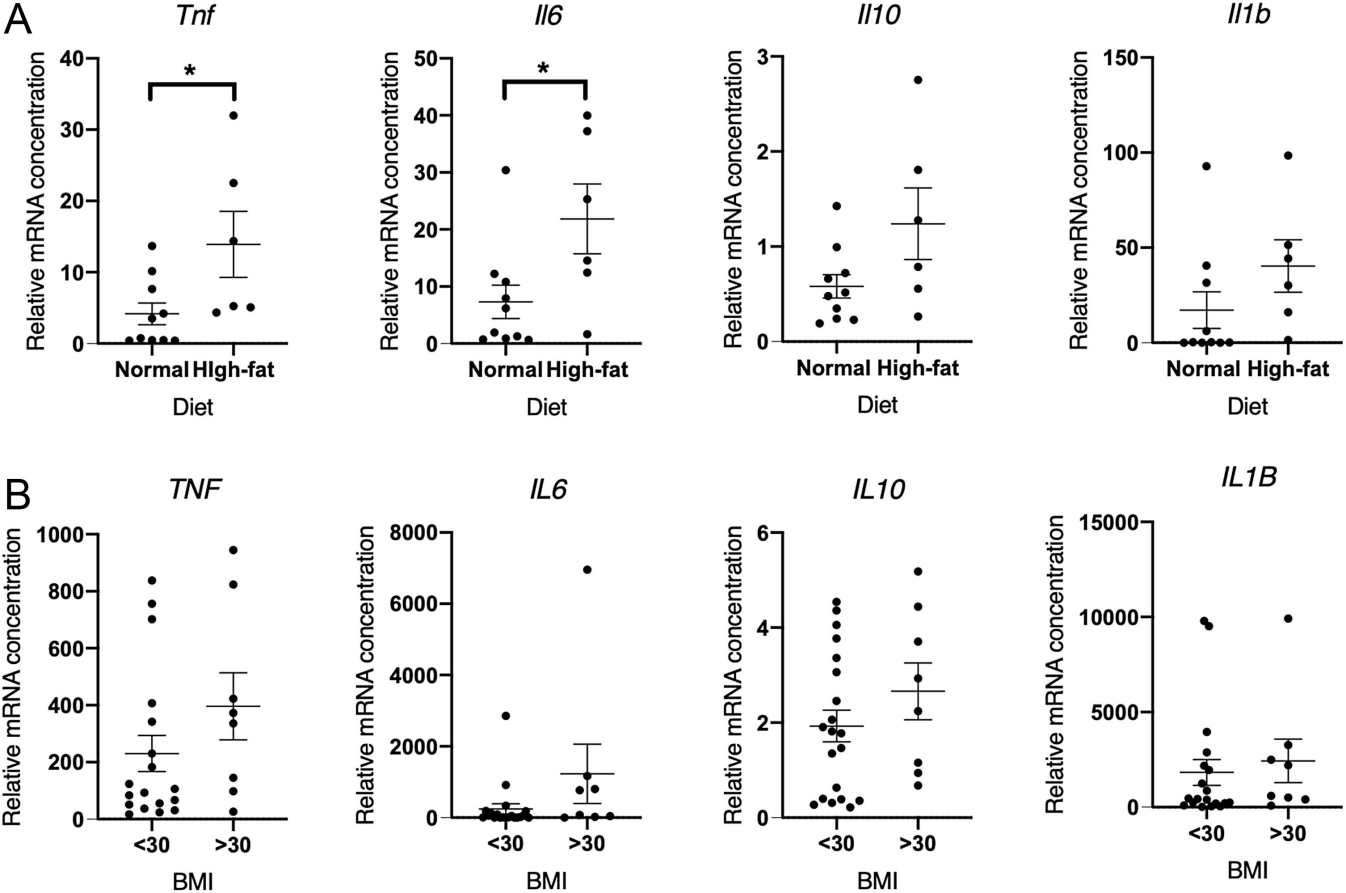
RT-qPCR assessment of inflammatory mediators. (A) *Tnf*, *Il6*, *Il10*, *Il1b* in decidualised uterine tissue from mice on normal (*n*  = 10) and high-fat (*n*  = 6) diets 24 h after progesterone withdrawal. (B) *TNF*, *IL6*, *IL10*, *IL1B* in endometrial tissue following progesterone withdrawal from women with normal (<30, *n*  = 20) and obese (>30, *n*  = 8) BMI measurements. *P < 0.05.

Increased inflammation of the endometrium at menstruation has previously been associated with heavy menstrual bleeding (Smith *et al.* 1981, Malik *et al.* 2006). We examined four recognised inflammatory mediators in the mouse uterine and human endometrial samples taken from women in the late secretory/menstrual phase without fibroids >3 cm. Mice on a high fat diet had significantly increased uterine *Tnf* and* Il6* when compared to mice maintained on normal diet (*P* < 0.05) (Fig. 6A). There were no significant differences in uterine *Il10* or *Il1b*, but both inflammatory mediators had a higher median value than mice on a high fat diet. Endometrium from women with obesity did not show significantly higher *TNF* or* IL6*, *IL10* or *IL1B* vs endometrium from women with a normal BMI (Fig. 6B).

## Discussion

In the present study, we found that the BMI of women was positively correlated with menstrual blood loss. Mice on a high fat diet had significantly increased body weight and delayed endometrial repair at simulated menstruation when compared to mice on a normal diet, consistent with prolonged menstrual bleeding. Examination of the uterus of these high fat diet mice 24 h following progesterone withdrawal (i.e. at the time of menstrual repair) revealed decreased luminal epithelial cell proliferation and increased local inflammatory mediators. These findings indicate that increased body weight impacts on endometrial function during menstruation resulting in increased menstrual blood loss.

Adipose tissue is a dynamic tissue with important metabolic and endocrine functions (Ouchi *et al.* 2011). It has a key role in metabolism of sex steroids and is an important source of oestrogen due to its aromatase activity, converting androgens to oestrone (Siiteri 1987). This has significant implications in post-menopausal women with obesity, providing unopposed oestrogens and increasing the risk of endometrial cancer (Onstad *et al.* 2016). Adipose tissue is also known to produce a number of adipokines, including leptin, adiponectin, resistin and plasminogen activator inhibitor-1 (Kershaw & Flier 2004). Adipose tissue functions are dysregulated in those with obesity (Ouchi *et al.* 2011), with aberrant secretion of adipokines altering inflammatory responses, endothelial cell function and coagulation.

Previous studies assessing the impact of obesity on endometrial function have focused mainly on endometrial cancer (MacKintosh *et al.* 2019) or implantation during assisted conception (Broughton & Moley 2017). To our knowledge, this is the first study of the impact of obesity on the volume of menstrual blood loss in regularly cycling women, where there is sequential exposure to oestradiol and progesterone. We found a weak positive correlation between BMI and menstrual blood loss assessed by PBAC score. This human data is limited by a number of factors. We assessed the amount of menstrual blood loss over one cycle and acknowledge that the volume of blood may vary in different cycles in the same woman. Our sample population had a high prevalence of HMB, with 63% having an estimated menstrual blood loss of >80 mL. Prevalence of HMB in the general population has been estimated at up to 33% (RCOG 2011), although lack of a robust definition that considers cultural, social and environmental influences means these figures may be difficult to interpret. However, caution should be applied in generalising the findings herein to non-gynaecology settings. We also acknowledge the heterogeneity of the women in our study and that BMI and menstrual blood loss have multiple risk factors. The lipid profile of our study participants was unknown. Although increased serum triglycerides are associated with endometrial cancer (Seth *et al.* 2012) the impact of lipid profile on the endometrium and menstrual physiology requires further research. Only one participant in our study had diabetes so we feel this factor would not have been a significant confounder. Multiple regression analysis for the presence of fibroids revealed that the correlation between BMI and menstrual blood loss remained close to statistical significance, indicating that there is still a strong suggestion of a BMI effect on menstrual blood loss independent of the effect of fibroids. However, other factors such as adenomyosis and the presence of coagulopathies (Munro *et al.* 2018) were unknown in our study population. Therefore, to minimise heterogeneity and delineate the role of body weight on endometrial function at menstruation, we utilised our mouse model of simulated menses. We observed reduced rates of decidualisation in response to the artificial stimulus of transcervical injection of oil, consistent with previously published findings in obese mice without hormone manipulation to induce menses. This previous study examined the impact of obesity on implantation and found that mice on a high fat diet had impaired decidualisation, with 50% smaller deciduomas in pseudopregnant mice and significantly smaller implantation sites in pregnant mice (Rhee *et al.* 2016). The mechanism for this reduced decidualisation rate in diet-induced obesity remains undetermined and is an area for future research. The impaired decidualisation rate seen in women with polycystic ovary syndrome has been linked to progesterone resistance (Piltonen *et al.* 2015) and it remains to be determined if obesity has a similar impact on endometrial progesterone response.

In the mice that underwent decidualisation, which is a prerequisite for menstruation, we observed significantly delayed endometrial repair 24 h after progesterone withdrawal in mice on a high-fat diet. This diet induced mouse model of obesity has been extensively used to study endocrine and inflammatory mechanisms in obesity (Lyall *et al.* 2020, Zheng *et al.* 2020). However, a limitation of this model is that it excludes non-dietary risk factors for obesity, for example, altered sleep patterns, lack of physical activity and endocrine disruption. This may be a potential limiting factor when translating results to humans. It is possible that the difference in endometrial repair between our two groups is not caused by diet, but is instead an artefact of differences in the decidualisation failure rate caused by the diet. However, our necessary exclusive inclusion of mice that have decidualised in the high-fat diet group is clinically relevant, as this is a similar selection process to our inclusion of women with a high BMI who have regular menstrual cycles, tha is, are likely to be ovulating regularly and therefore undergo spontaneous decidualisation in the secretory phase. Women with ovulatory dysfunction should be treated using different clinical protocols to those presenting with regular cycles. Our findings in this mouse model are consistent with our finding of a positive correlation between BMI and MBL in regularly cycling women. Examination of endometrial proliferation revealed decreased luminal epithelial BrdU staining in mice on a high-fat diet. Luminal reepithelialisation is an essential part of repair of the denuded endometrial surface at menstruation (Garry *et al.* 2009) and reduced proliferation may contribute to prolonged menstrual bleeding. A potential explanation for this decreased proliferation may be the altered effects of leptin with increased body weight. Leptin is a hormone secreted from adipose cells that regulates energy balance by suppressing food intake. Obesity has been shown to cause leptin resistance (Klok *et al.* 2007). A human endometrial epithelial cell line exposed to physiological levels of leptin displayed increased proliferation* in vitro*, leading authors to propose that leptin has an important role in endometrial remodelling (Tanaka & Umesaki 2008). Hence leptin resistance in those with obesity may result in decreased endometrial proliferation and could contribute to delayed endometrial repair at menstruation.

We have shown that endometrial hypoxia is necessary for normal endometrial repair in our mouse model of simulated menstruation (Maybin *et al.* 2018). Therefore, we examined a panel of known hypoxia regulated genes to determine if there was an altered endometrial hypoxic response at menstruation in those with obesity. Data herein show a non-significant trend towards increased hypoxia-regulated genes in endometrium from high fat diet mice and from women with a BMI >30. We acknowledge that our numbers for these studies in mouse uterus and human endometrial tissue are relatively small and there is variability in our outcome measurements, likely due to the heterogeneity and complexity of examining *in vivo* tissue. This may account for the lack of statistical significance. This non-significant increase in hypoxia regulated factors was unexpected and may be due to relative low numbers but could also be explained by increased or prolonged endometrial hypoxia during menstruation in those women with obesity and mice on a high-fat diet. Alternatively, the physiological hypoxia of menstruation that is observed 8 h following progesterone withdrawal in the mouse model (Cousins *et al.* 2016, Maybin *et al.* 2018) may be delayed until 24 h in mice with increased body weight. The role of hypoxia in obesity is debated in the literature. Mouse studies suggest hypoxia in adipose tissue has an important role in the adipose tissue dysfunction observed in obesity, but this has not been confirmed in human studies (Goossens & Blaak 2015). The impact of any such adipose tissue hypoxia on end organ function remains to be determined.

When functioning normally, adipose tissue produces multiple factors that result in pro- and anti-inflammatory effects. However, obesity is associated with dysfunctional adipose tissue, characterised by macrophage infiltration (Ouchi *et al.* 2011). This infiltration results in a more pro-inflammatory profile with obesity-induced inflammation causing disorders at other tissue sites, including the pancreas (Hotamisligil *et al.* 1993, Saltiel & Olefsky 2017). Our findings herein are consistent with these observations at other tissue sites, detailing increased local inflammatory mediators in the uterus of mice on a high fat diet. A previous study of human endometrial tissue found no significant association between BMI and altered endometrial gene expression measured by RNASeq (Holdsworth-Carson *et al.* 2020). Consistent with these findings in women with and without endometriosis, none of the inflammatory mediators examined herein were significantly increased in endometrium from women with obesity vs normal BMI. However, all showed non-significant increased levels in the presence of obesity. This lack of significance may reflect the small sample number and the inevitable human tissue heterogeneity due to differences in parity, age and hormone levels. Most of this variability can be overcome by utilising the mouse model of simulated menses. Given the known heterogeneity of human participants and our findings in the mouse model of simulated menses, overall these data are consistent with a pro-inflammatory peri-menstrual endometrial environment in the presence of increased adiposity. Women with abnormal uterine bleeding have been shown to have an increased inflammatory response in their endometrium at menstruation (Malik *et al.* 2006, Smith *et al.* 2007). In particular, TNF has been implicated in the aetiology of HMB, with studies demonstrating increased levels of TNF in the menstrual effluent of women with HMB compared to normal controls (Malik *et al.* 2006). Therefore, this pro-inflammatory profile of endometrium during menstruation may contribute to increased blood loss in women with obesity.

The impact of obesity on endometrial function is thus likely to be multifactorial. Our data have revealed that increasing BMI is positively correlated with menstrual blood loss in women and have confirmed that a high fat diet significantly delayed endometrial repair in a mouse model of menstruation. Mice with increased body weight also displayed significantly elevated uterine inflammatory mediators and decreased endometrial epithelial cell proliferation. The impact of obesity on menstrual blood loss has been scarcely reported in the literature and we believe our results provide new evidence that increased body weight may contribute to heavy menstrual bleeding. This will facilitate evidence-based shared decision making between clinicians and patients regarding lifestyle adjustments in the management of this common symptom.

## Declaration of interest

J J R, C W, A M, S B-M, S S, M N, A C and J A M have no conflicts of interest to declare. H O D C has clinical research support for laboratory consumables and staff from Bayer AG and provides consultancy advice (but with no personal remuneration) for Bayer AG, PregLem SA, Gedeon Richter, Vifor Pharma UK Ltd, AbbVie Inc; Myovant Sciences GmbH. H O D C receives royalties from UpToDate for article on abnormal uterine bleeding.

## Funding

This work was supported by Wellcome Trust Fellowships 209589/Z/17/Z and 100646/Z/12/Z, Wellbeing of Women grant RG1820 and the Barbour Watson trust. This work was also in part funded by MRC Research Grants G0000066, G0500047, G0600048, and MR/ J003611/1. The work was undertaken in the MRC Centre for Reproductive Health, funded by grants G1002033 and MR/N022556/1.
